# Characterization of the Pathogenesis of H10N3, H10N7, and H10N8 Subtype Avian Influenza Viruses Circulating in Ducks

**DOI:** 10.1038/srep34489

**Published:** 2016-09-28

**Authors:** Miaomiao Zhang, Xingxing Zhang, Kaidi Xu, Qiaoyang Teng, Qinfang Liu, Xuesong Li, Jianmei Yang, Jianqing Xu, Hongjun Chen, Xiaoyan Zhang, Zejun Li

**Affiliations:** 1 Shanghai Public Health Clinical Center, Fudan University Shanghai 201508 P. R. China; 2 Shanghai Veterinary Research Institute, Chinese Academic of Agricultural Sciences, Shanghai 200241 P. R. China; 3 Animal Influenza Virus Evolution and Pathogenesis Innovation Team of The Agricultural Science and Technology Innovation Team, Shanghai 200241 P. R. China.

## Abstract

Three H10 subtype avian influenza viruses were isolated from domestic ducks in China, designated as SH602/H10N8, FJ1761/H10N3 and SX3180/H10N7, with an intravenous pathogenicity index (IVPI) of 0.39, 1.60, and 1.27, respectively. These H10 viruses showed a complex pathology pattern in different species, although full genome characterizations of the viruses could not identify any molecular determinant underlying the observed phenotypes. Our findings describe the pathobiology of the three H10 subtype AIVs in chickens, ducks, and mice. FJ1761/H10N3 evolved E627K and Q591K substitutions in the gene encoding the PB2 protein in infected mice with severe lung damage, suggesting that H10 subtype avian influenza viruses are a potential threat to mammals.

H10 subtype influenza viruses have been isolated in various species of waterfowl across worldwide geographic areas for more than 50 years^1^,^2^,^3^. The viruses remain avian receptor binding, however, some strains are highly pathogenic to chickens, even though they lack multiple basic amino acids at the hemagglutinin cleavage site^4^,^5^,^6^,^7^. H10 viruses occasionally infect humans. An H10N3 virus was isolated in Hong Kong in 1979^8^, and in a live-bird market in Thailand in 2011^9^. However, pathogenicity in mammals due to H10N3 viruses remains largely unclear. The first H10N7 isolate was identified in chickens in Germany^10^. In 2010, an H10N7 strain caused disease in a chicken farm in Australia^11^. Recently, an H10N7 virus was isolated from dead harbor seals in Denmark^12^. A novel reassortant H10N7 AIV was found in chickens in Eastern China^11^,^12^,^13^,^14^,^15^,^16^,^17^,^18^,^19^,^20^,^21^,^22^,^23^. Additionally, an H10N4 isolate caused an outbreak of respiratory disease in mink in Sweden^15^. H10N5 virus was detected in pigs in 2008^24^.

Human infections with H10N8 subtype avian influenza virus (AIV) were reported in Jiangxi province, China, in 2013–2014^25^. Sequencing these viruses showed that all six internal segments were from the H9N2 subtype G57 genotype^26^. Transmission of this subtype from avian species to humans increases the risk of adaptive point mutations or reassortment events with H7N9, H9N2 subtype AIV, or human seasonal viruses, which could be the source of a highly pandemic virus^27^,^28^. The H10N8 virus also showed high pathogenicity in mice^29^,^30^. A subsequent surveillance study also showed the presence of H10N8 in waterfowls, feral dogs, and live poultry markets (LPMs)^26^,^27^,^31^,^32^. While multiple H10 genotype viruses (e.g. H10N8, H10N3, and H10N7) are circulating in LPMs in China, their potential to infect mammals remains largely unknown. To address this question, three H10N8, H10N7, and H10N3 subtype influenza viruses circulating in domestic ducks were characterized in this study. We found that their complex reassortments and pathobiology patterns in chickens, ducks, and mice indicates a potential threat to humans.

## Results

### Complex reassortment patterns of the three H10 subtype influenza viruses

Three strains of H10 subtype avian influenza virus were isolated from healthy domestic ducks in different provinces of China (Table 1). The isolates were designated as A/duck/Shanghai/602/2009 (H10N8) (thereafter SH602/H10N8), A/duck/Fujian/1761/2010 (H10N3) (thereafter FJ1761/H10N3), and A/duck/Shanxi/3180/2010 (H10N7) (thereafter SX3180/H10N7).

To characterize the molecular evolution of the three H10 viruses, the full-length genomes of the serially purified H10 viruses were sequenced and analyzed by using RT-PCR (Table 1).

**Figure 1:**
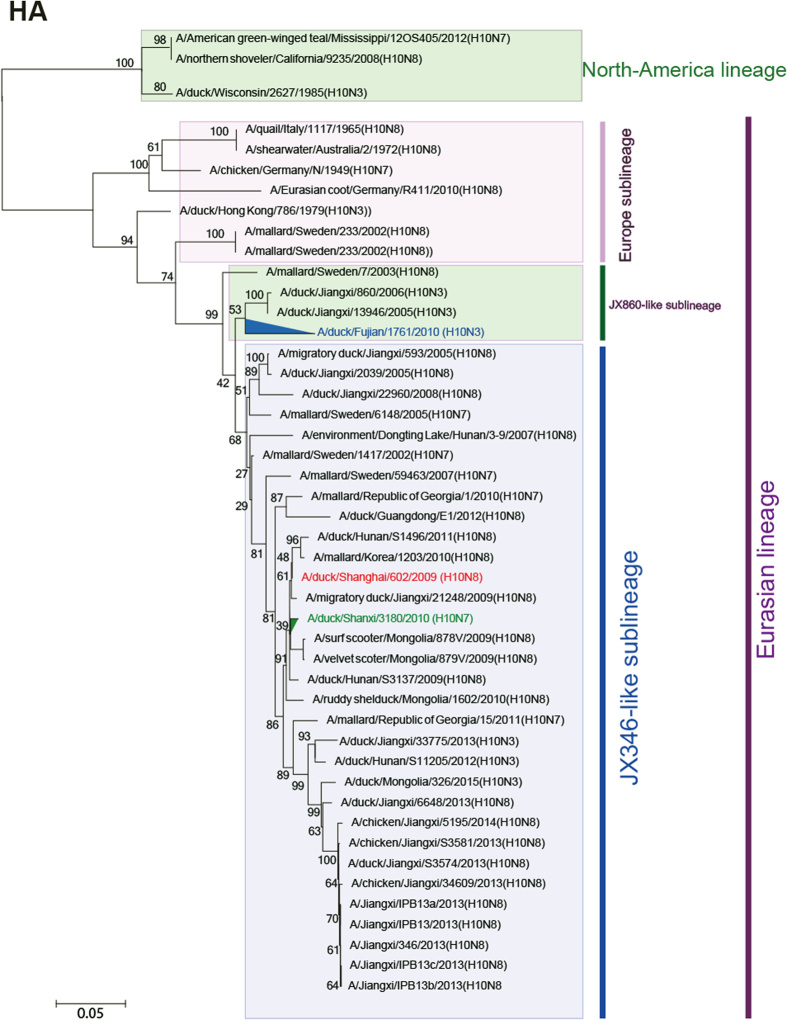
Phylogenetic tree of HA and NA sequences of H10 subtype AIVs. The phylogenetic tree was generated with MEGA6 software, which was based on the complete sequence of HA sequences (**A**), NA sequences (**B**) and PB2 (**C**). The reliabilities of the phylogenetic trees were assessed by bootstrap analysis with 1, 000 replications. Different colors for each segment represent supposed reassortment patterns for the H10 viruses.

In the phylogenetic tree of HA sequences, these viruses comprise different sublineages of the Eurasian lineage. H10N3 fell in the Europe sublineage, and H10N7 and H10N8 were located in the JX346-like (Asian) sublineage, which also contains H10N8 viruses (Fig. 1A). The three H10 isolates shared the amino acid sequence (PEIMQGRGLFG) at the cleavage site between HA1 and HA2, indicating they are low pathogenic strains. The amino acids 95Y, 151W, 183H, 190E, 191K, 194L, 226Q, 227S, 228G, and 229R were observed at the receptor-binding pocket area of all 3 viruses. None of these residues have been reported to be involved in the recognition of human-type receptors, suggesting that all the isolates likely bind to avian-like receptors^30^.

All the isolates are likely susceptible to neuraminidase inhibitors (Oseltamivir, Zanamivir, and Peramivir) based upon their NA amino acid sequences^33^. In the phylogenetic trees of NA genes, evolution of the three strains showed significant differences (Fig. 1B). SH602/H10N8 reassorted with a strain from an American lineage, closely related to A/duck/Beijing/33/04 (H3N8)^25^. FJ1761/H10N3 reassorted with A/duck/Zhejiang/12/2011 (H7N3), which has been classified in the Eurasian lineage^34^. SX3180/H10N7 reassorted with A/mallard/Netherlands/2/2009 (H7N7) in the Eurasian lineage.

The PB2 segment of FJ1761/H10N3 seems to be derived from a highly pathogenic H5N1 strain (Fig. 1C). However, the PB2 segments of SH602/H10N8 and SX3180/H10N7 viruses might be derived from different H4N6-like strains isolated from Mongolia or China, respectively (Fig. 1C).

For PB1 and PA, FJ1761/H10N3 virus showed a unique reassortment pattern, in that the PB1 and PA segments were not from H4N6 subtype viruses (Supplementary Fig. 1A,B), but were instead derived from an H7N3 subtype AIV in Eastern China, very closely related to A/duck/Zhejiang/12/2011 (H7N3), which is also a donor for H7N9 AIV in humans^34^. For the NP segment, SX3180/H10N7 and FJ171/H10N3 viruses fell into an H7N3-like group, but only NP of SH602/H10N8 was from H4N6 subtype AIV (Supplementary Fig. 1C). The M and NS segments of all the three viruses appear to originate from a Korean H4N6-like subtype AIV isolated from wild ducks (Supplementary Fig. 1D,E)^35^.

Amino acids E627 and D701 were found in PB2 of all three H10 isolates, which suggests that the 3 isolates are poorly adapted to mammals^29^. Amino acids L26, V27, A30, and S31 in the M2 protein confer no resistance to M2 ion channel drugs^36^,^37^. The three H10 viruses bear an ESEV motif in their NS1 carboxy termini,. indicating an H5N1-like PDZ domain related to virulence^38^.

### The pathogenicity of H10 viruses vary in chickens

To determine the pathogenicity of the H10 viruses in chickens, the virus stocks were purified three times by end-point infection. All three H10 viruses replicated to high titers in eggs (Table 1). The viruses were injected into the veins of chickens and 10 days later the intravenous pathogenicity indices were calculated. The H10 viruses varied in pathogenicity to chickens. SH602/H10N8 is a lentogenic strain with an IVPI value of 0.39, and FJ1761/H10N3 and SX3180/H10N7 are highly pathogenic to chickens with IVPI values of 1.60 and 1.27 respectively^39^ (Table 1).

**Figure 2:**
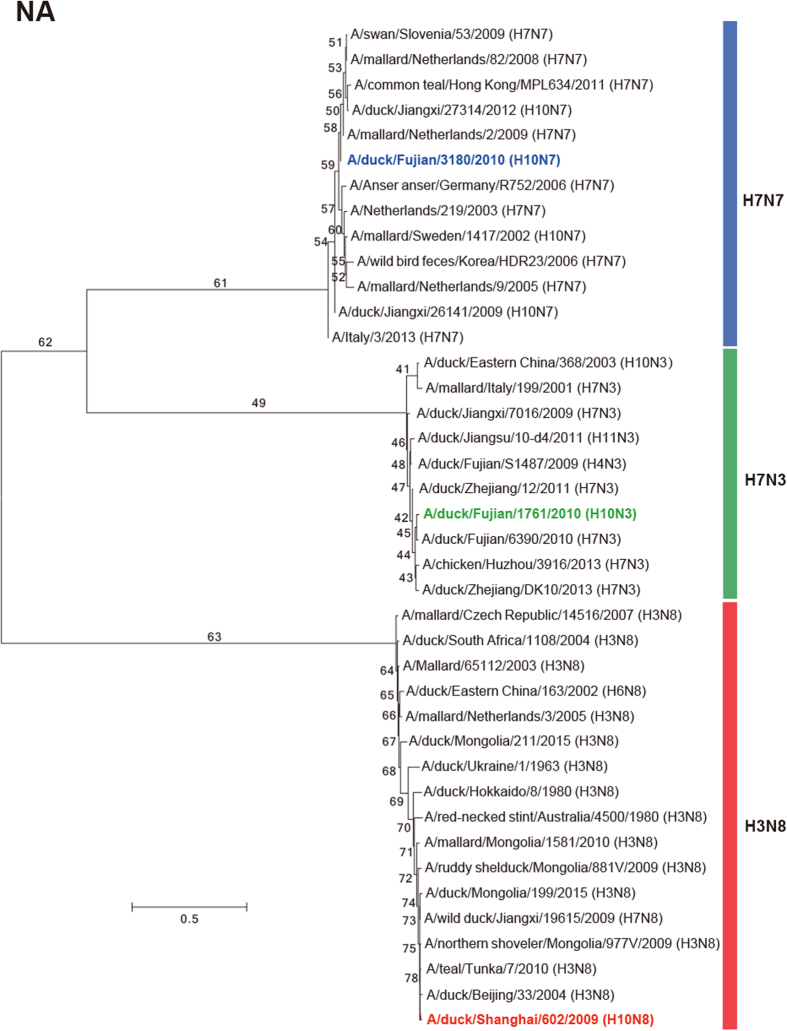
Virulence of the H10 viruses in chickens. (**A**) SPF chickens of each group (*n* = 10) were intranasally inoculated with 100 μl virus stocks at a dose of 10^6 ^EID_50_ and 4-week-old uninfected chicken (*n* = 6) were introduced at 2 dpi as direct contacts. The titration of all the oralpharyngeal and the cloaca swabs were detected. (**B**) The sera of the chickens were collected for HI test at 14 dpi.

To characterize further the virulence and transmissibility of the H10 viruses in chickens, 4-week-old SPF chickens (10/group) were intranasally inoculated with 100 μl virus stocks at a dose of 10^6 ^EID_50_. The titers of all the oralpharyngeal and the cloaca swabs in the SH602/H10N8 virus groups were below the detection limit at 3 dpi. However, a sight difference was observed between the FJ1761/H10N3 and SX3180/H10N7 viruses. In FJ1761/H10N3 virus - infected chickens, 3/10 oralpharyngeal swabs were positive with low titers of 10, 178, and 316 EID_50_/ml. In the cloaca swabs for this virus, only one was positive with a low titer of 316 EID_50_/ml. A low transmission capability of FJ1761/H10N3 was observed found in one of six oralpharyngeal swabs in the contact group (FJ1761-C group) with a low titer (10 EID_50_/ml). No virus was found in the cloaca swabs of the FJ1761-C group. For SX3180/H10N7-infected chickens, one was positive with a high titer of 1.78 × 10^3^ EID_50_/ml and another was positive without dilution (10 EID_50_/mL). No virus was found in the contact group (SX3180-C) (Fig. 2A).

At 3 and 5 dpi, three chickens from each group were euthanized. No lesions were observed. No viruses were found in the chicken lungs by either titration or RT-PCR analysis. No significant pathology was observed in the lungs after H & E staining. The sera of the remaining chickens were collected for hemagglutination inhibition (HI) assays at 3, 5, and 14 dpi. Except for the titers under the detection limit at 3 dpi and 5 dpi, the sera were positive at 14 dpi. Three of four serum samples were positive with titers of 64, 128, and 128 in the SH602-I group, but the sera of the SH602-C group were under the detection limit. In the FJ1761-I group, all the HI titers were positive with titers of 32, 64, 128, and 256. In the FJ1761-C group, three sera samples were positive with titers of 128. In the SX3180-I group, the HI titers were also positive with HI titers of 32, 64, 128, and 256. In the SX3180-C group, three sera samples were positive with titers of 32, 64, and 128. Thus, FJ1761/H10N3 and SX3180/H10N7 viruses infect and are transmitted between chickens, whereas SH602/H10N8 virus does not (Fig. 2B).

### The H10 viruses were avirulent but transmissible in ducks

**Figure 3:**
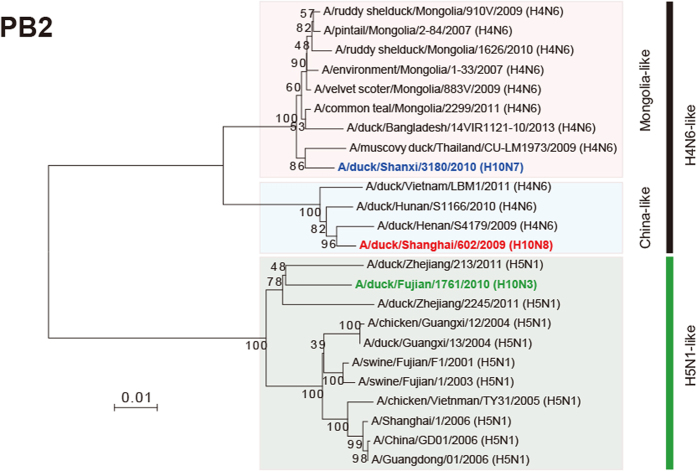
Pathogenesis and transmission of the H10 viruses in ducks. (**A**) Ducks (*n* = 10) were intranasally inoculated with 100 μl viruses at a dilution of 10^6 ^EID_50_ for each group and 4-week-old uninfected ducks (*n* = 6) were introduced at 2 dpi as the direct contacts. The titers of all the oralpharyngeal and the cloaca swabs were detected. (**B**) The oralpharyngeal and cloaca swabs at 5 dpi. (**C**) The remaining ducks were euthanized at 14 dpi and the sera were collected for HI test.

At 3 dpi of oralpharyngeal swabs, 70% of samples in SH602/H10N8-infected ducks (SH602-I group) were positive but with low titers (<20 EID_50_/ml), in which the highest titer was 316 EID_50_/ml. Four out of six oralpharyngeal swabs in the SH602-C group were positive. For the FJ1761-I group at 3 dpi, 50% oralpharyngeal swabs were positive, but with titers less than 50 EID_50_/ml, in which the highest titer was 316 EID_50_/ml. However, the virus was not detected in the oralpharyngeal swabs of the FJ1761-C group. For SX3180/H10N7, only one sample was positive with a titer of 178 EID_50_/ml. No virus was detected in the oralpharyngeal swabs of SX3180-C group (Fig. 3A).

A greater percentage of cloacal swabs were positive at 3 dpi, suggesting that the viruses may be transmitted by the fecal-oral route. In the SH602-I group, 100% of samples were positive but at titers less than 100 EID_50_/ml, in which the highest titer was 1.78 × 10^3 ^EID_50_/ml. All samples in the SH602-C group were positive (<100 EID_50_/ml), in which the highest titer was 562 EID_50_/ml. The titers of the FJ1761-I group at 3 dpi were higher than in the SH602-I group; 100% of oralpharyngeal swabs were positive with a mean titer of 282 EID_50_/ml, in which the highest titer was 1.78 × 10^4 ^EID_50_/ml. All swabs of the FJ1761-C group were positive with a mean titer of 10^2.13 ^EID_50_/ml, in which the highest titer was 3.16 × 10^3 ^EID_50_/ml. For SX3180/H10N7, 50% of samples were positive but at titers less than 100 EID_50_/ml. Two out of six samples were positive in the oralpharyngeal swabs of the SX3180-C group (Fig. 3A).

The titers of the H10 viruses were lower in oralpharyngeal swabs at 5 dpi. Only one of seven samples was positive in the SH602-I and FJ1761-I groups, two of six samples were positive in the SH602-C group, and four of six samples were positive in the FJ1761-C group. Two of seven samples were positive in the SX3180-I group and three of four samples were positive in SX3180-C group (Fig. 3B).

However, the virus titers of cloaca swabs increased at 5 dpi. Five of seven samples were positive in the SH602-I, FJ1761-I, and SX3180-I groups with mean titers of 501, 112, and 79 EID_50_/ml, respectively. Five of six samples were positive in the SH602-C group with the higher titer of 1.12 × 10^3 ^EID_50_/ml, and 100% samples were positive with titers of 135 and 380 EID_50_/ml for FJ1761-C and SX3180 group, respectively (Fig. 3B).

The remaining seven ducks, including four inoculated and three contact ducks in each group were monitored daily for clinical signs. All survived the 14-day observation period. The ducks were euthanized at 3, 5 and 14 dpi and serum was collected for HI test. HI titers at 14 dpi were less than 32 in all groups (Fig. 3C), No positive HI reactions were observed for sera collected at 3 and 5 dpi.

### The three H10 viruses showed low virulence in Balb/c mice

**Figure 4:**
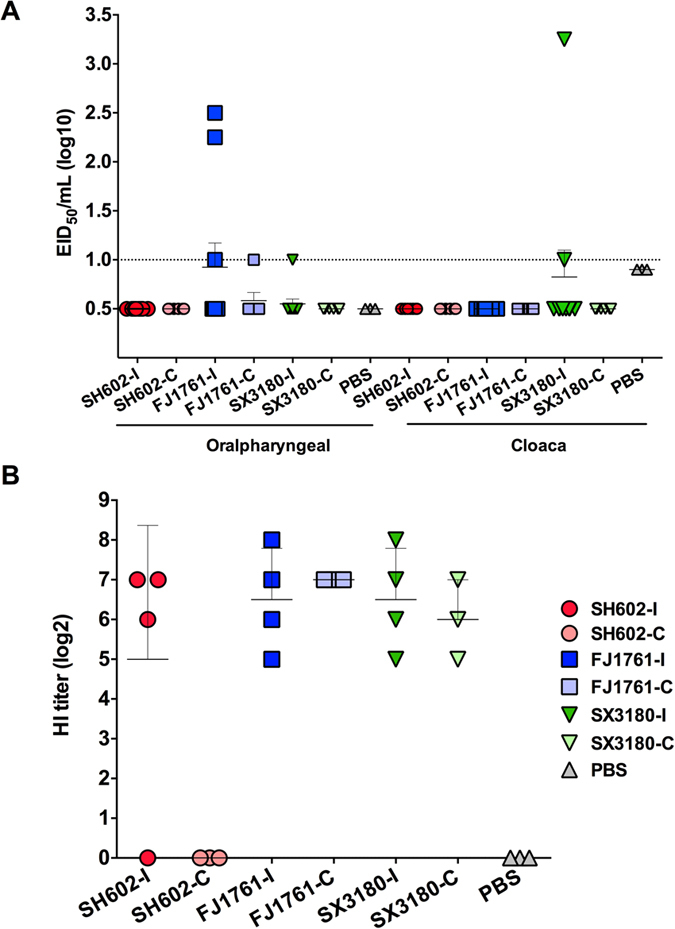
Virulence of the H10 viruses in mice. Virus levels detected in lung samples and nasal turbinates of mice at 3 dpi (**A**) or 5 dpi (**B**). Mouse body weight changes are shown (**C**).

To determine the virulence of these H10 viruses in mice, each mouse was inoculated with 50 μl of diluted viruses at a dose of 10^6 ^EID_50_. All mice survived until 10 dpi (Fig. 4C). HI titers were undetectable at all time points assayed.

At 3 dpi, the mice had a lung titer of 5.62 × 10^5 ^EID_50_/g in the SH602/H10N8 group, much higher than that in the FJ1761/H10N3 and SX3180/H10N7 groups (Fig. 4A; p<0.01). At 5 dpi, the virus titers were similar (Fig. 4B). FJ1761/H10N3 replicatated in nasal turbinates to a titer of 4.78 × 10^3 ^EID_50_/g, which was much higher than the other groups (p<0.01). Compared to the negative control challenged with 0.01M phosphate-buffered saline (PBS) buffer, all three H10 viruses induced a slight body weight loss of less than 5% (Fig. 4C).

**Figure 5:**
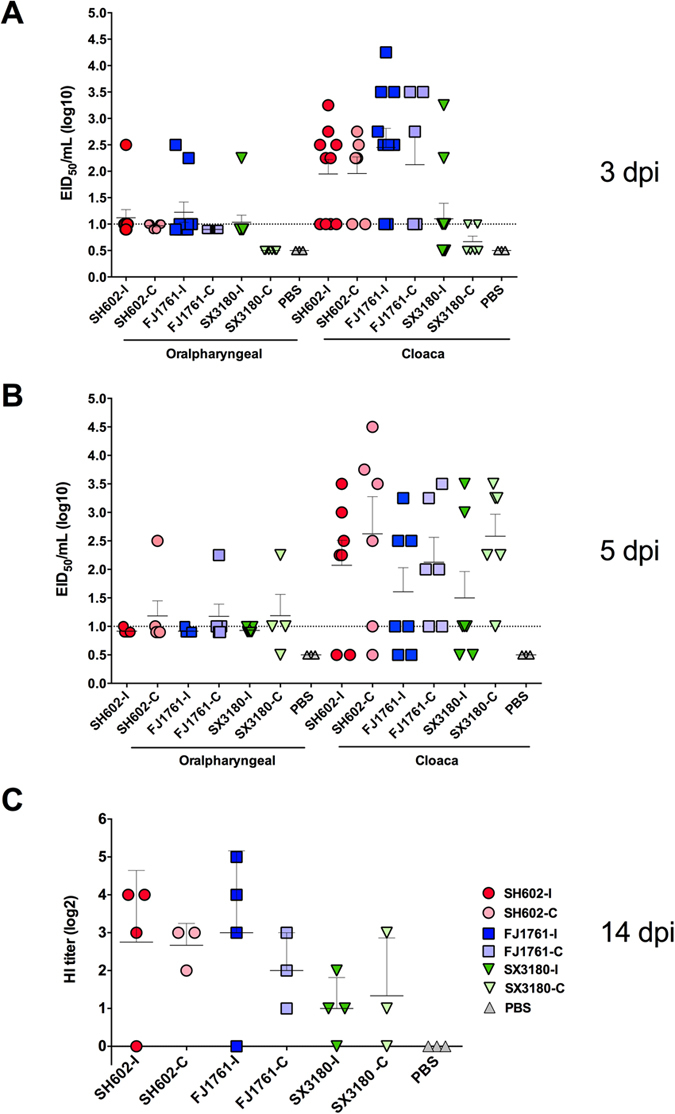
Representative histopathological changes of mice after H10 virus infections. (**A**) Control mice. (**B**) Mice infected with SH602/H10N8 virus showed minimal pathology in the lungs. (**C**) Mice infected with SX3180/H10N7 virus. Arrows indicate diffuse pneumonia. (**D**) Mice infected with FJ1761/H10N3 virus. Arrows indicate classical acute lung injury.

On day 3, mice infected with SH602/H10N8 showed minimal pathology in the lungs (Fig. 5B). FJ1761/H10N3 and SX3180/H10N7 viruses showed a strong inflammatory response. For the mice infected with SX3180/H10N7, the lung lesions were characterized by diffuse pneumonia, thickening of the alveolar wall, shedding of the bronchial epithelium, slight infiltration of neutrophils in the bronchioles, and peribronchiolar and vascular edema and hemorrhaging (arrows in Fig. 5C). The lung samples of the FJ1761/H10N3-infected mice were characterized by classical acute lung injury, which showed peribronchiolar lesions and bronchiolitis, interstitial and alveolar edema, inflammatory cell infiltration around small blood vessels, and thickening of alveolar walls (Fig. 5D, arrows). Control mice had no apparent histological changes (Fig. 5A).

Sanger sequencing of the PB2 segments of viruses isolated from the lung samples revealed that two FJ1761/H10N3 isolates had either a Q591K or E627K substitution. No mutations were detected in the third FJ1761/H10N3 isolate or in any isolate from the other two viruses (Fig. 5D). E627K and Q591K substitutions may thus be important mammalian-adaption mutations, as indicated in previous studies^29^,^40^.

## Discussion

Low pathogenic AIVs do not cause explicit symptoms in chickens or waterfowls^31^. In the last two decades, interspecies transmissions of these viruses to humans has occurred frequently^41^. During the spring of 2015, the World Health Organization (WHO) reported 132 human H7N9 infections with 44 deaths. However, H7N9 subtype AIV isolates do not possess a classical highly pathogenic phenotype^3^.

LPAIV strains are endemic to numerous host species, and many antigenically distinct strains co-circulate, such as H3N8 and H4N6 AIV isolated from domestic ducks, which play a central role in influenza persistence and reassortment^42^. As 65% of the global population of ducks are bred in China, this species formed the major source of influenza viruses to humans or poultry. In our study, compared to the location of HA sequences from SH602/H10N8 and SX3180/H10N7 viruses, FJ1761/H10N3 virus was found in a different sublineage in the Eurasian lineage (Fig. 1). The H10 subtype, in combination with various N subtypes, was previously thought to occur mainly in avian strains. For the NA segment, SH602/H10N8 virus was reassorted with an H3N8 strain from an American lineage. FJ1761/H10N3 virus was reassorted with an H7N3 virus, which was also found in human H7N9 viruses^34^. SX3180/H10N7 virus was reassorted with an H7N7 virus sublineage from the Eurasian lineage. Alignments of internal genes were more complex, with the three viruses appearing to be derived from H4N6 or H7N3 subtype AIVs. Especially, PB2 segment from FJ1761/H10N3 virus was highly homologous to the highly pathogenic H5N1 AIV from Eastern China (Fig. 1C). PB2 had the classical mutation at 627 aa (Fig. 5D), which also suggested a potential threat to humans or poultry^35^.

The three viruses had differing degrees of virulence in chickens (Table 1). Hospital surveillance of patients with severe pneumonia found a novel H10N8 AIV in Jiangxi, China^25^. The H7N9 and H10N8 viruses in humans were reassorted in a similar pattern. All the six internal segments were derived from H9N2 viruses in chickens and its surface proteins were similar to viruses found in domestic ducks and wild birds^27^. Since the internal segments of the three isolates were not from H9N2 viruses (Fig. 1), whether these H10 viruses could infect or/and transmit among mammals or poultry is unclear.

Although challenged ducks did not show significant lesions in all tissues, oralpharyngeal and cloaca swabs showed different levels of virus shedding (Fig. 3). Thus, the H10 viruses can replicate and circulate in domestic ducks by the fecal-oral route. The mechanisms of influenza virus replication and their interaction with the innate immune system are current areas of investigation^43^,^44^. H10 viruses varied in their pathogenicity and their transmission in chickens or mammals, although they did transmit in ducks (Fig. 2).

In 1984, the H10 subtype, in combination with an N4 influenza epidemic, occurred on Swedish mink farms, designated as mink/84^45^. The *ns* gene of this strain appears to have contributed to the virulence of the virus in mink by helping the virus evade innate immune responses^46^. Viruses of avian H1, H6, H7, H10, and H15 subtypes cause severe disease in mice and damage human lung cells^47^. H10 viruses interact weakly with human-like receptors and maintain a strong affinity for avian-like receptors^48^, however, the three H10 viruses isolated in this study replicated in mouse lungs without prior adaptation (Fig. 4). The amino acid substitution from E to K at site 627 of the PB2 gene of FJ1761/H10N3 virus is the first step in virus adaptation in mammals and this substitution is host-dependent^29^. Compared to the three H10 viruses, we found that the PB2-E627K substitution significantly enhanced the pathogenicity of the H10N3 virus in one mouse and the PB2-Q591K substitution also slightly enhanced the virulence of the virus in another mouse. This mutation was found to contribute to mammalian pathogenesis for H9N2^49^, H7N9^50^,^51^, and H5N2^52^,^53^.

Taken together, from the complicated reassortant patterns and the complex virulence and transmission data for the three H10 viruses, we found these viruses caused no lesions to chickens or ducks, however, they might circulate in ducks and transmit in chickens, in which they could reassort with other subtypes. This might lead to the potential emergence of pandemic influenza virus in mammals.

## Methods

### Ethics statement

All animal studies were performed in strict accordance with the recommendations in the Guide for the Care and Use of Laboratory Animals of Shanghai Veterinary Research Institute, CAAS (ID: SHVRI-PO-2014-0098) and all animal research was approved by the Animal Association of Science and Technology Commission of Shanghai Municipality, China (Permit Number: 2013–11). Ten-day-old specific pathogen-free (SPF) chicken embryos were obtained from Merialvital Co. (Beijing, China).

### Viruses and cells

During the routine surveillance from 2009 to 2010, 192 out of 4, 000 oropharyngeal swabs collected from domestic ducks, geese, and chickens were positively identified as influenza A virus according to results from hemagglutination assays (HA) and real-time RT-PCR amplification of M segments^39^. Three H10 viruses were chosen for further study here. All the viruses were grown in specific pathogen free (SPF) 9-days old embryonated chicken eggs at 37 °C for 48 h. The allonatic fluid was collected in vials and stored into −80 °C until use. The viral titers were determined and calculated according to the Reed-Muench method for 50% egg infectious dose (EID_50_) or 50% tissue culture infectious dose (TCID_50_) on MDCK cells with minimum essential medium (MEM) with 2% bovine serum albumin (BSA) and 1 ug/ml trypsin treated with L - (tosylamido-2-phenyl) ethyl chloromethyl ketone (TPCK).

### Sequencing and phylogenic analysis of the H10 viruses

To understand the genetic character of the three viruses, the whole genomes of the isolates were sequenced by RT-PCR. The vRNAs and cDNAs from the allonatic fluids of the three viruses were prepared as previously described^54^. Briefly, total RNAs were extracted by using the RNeasy kit (Qiagen Inc, Gaithersburg, MD) following manufacturer’s instructions. Reverse transcription was carried out with the Uni12 primer (5′-AGCAAAAGCAAGG-3′) and avian myeloblastosis virus (AMV) reverse transcriptase (Promega, Madison, WI). The cDNAs were stored at −80 °C until use. The segments were amplified by PCR with the H10Nx universal primers using Phusion high-fidelity PCR master mix (New England Biolabs, Ipswich, MA)^54^. The PCR products and the ligated plasmids, in which the eight segments were subcloned into the pHW2000 vector, were sequenced by the Sanger method (GENEWIZ, Suzhou, China). The complete genomes of the three viruses are shown in Table 1 and then HA segments were analyzed and aligned by using MEGA6.

### Intravenous pathogenicity index (IVPI) in chickens

The viruses were subjected to IVPI tests following the *WHO Manual on Animal Influenza Diagnosis and Surveillance*^39^. In brief, ten 6-week-old chickens were inoculated with 0.1 ml 1:10 diluted viruses via intravenous route and two chickens were inoculated with 0.01 M PBS as a control group. Clinical signs were observed daily over ten days. At each observation, each chicken was scored 0 (normal), 1 (sick), 2 (severely sick), and 3 (dead). The clinical assessment of sick and severely sick chickens include ‘sick’ chickens would show one of the following signs and ‘severe sick’ more than one of the following signs: respiratory sign, depression, diarrhea, cyanosis of the exposed skin or wattles, edema of the face and/or head, or nervous signs. The IVPI index is the mean score per chicken per observation over the 10-day period^39^. The IVPI experiments and all the following animal experiments were approved by the Ethics and Biosafety Committee of Shanghai Veterinary Research Institute.

### Pathogenicity and transmission of H10 viruses in chickens

To identify the pathogenicity and transmissibility of H10 viruses, three groups of ten 4-week-old SPF chickens for the three H10 viruses were intranasally inoculated at an equal dose of 1 × 10^6 ^EID_50_ per 100 μl diluted in 0.01 M PBS buffer. Ten chickens were inoculated with 100 μl PBS as negative control. At the second day post-infection (dpi), six 4-week-old uninfected chickens were introduced into each group as direct contacts. The swabs from oralpharyngeal and cloaca samples in the groups above were collected everyday and then stored at −80 °C into 1 ml PBS containing 100 U/ml penicillin and 0.05 mg/ml streptomycin under sterile conditions.

Three chickens from each challenge group were euthanized randomly at 3 dpi and 5 dpi. Lung, spleen, trachea, kidney, pancreas, and brain samples were collected and homogenized using a Tissue Lyser apparatus in 1 ml PBS under sterile conditions. Three of the direct contacts in each group were euthanized at 5 dpi. Oralpharyngeal and cloaca samples were also collected in PBS buffer. The solid debris was pelleted by centrifugation at 12, 000 rpm for 10 minutes, and the homogenates were used for virus titration in 9-day-old SPF embryonated chicken eggs. The remaining chickens were monitored daily for clinical signs, euthanized at 14 dpi, and the sera was collected for HI assays. The HI assay was conducted according to the WHO manual on animal Influenza diagnosis and surveillance^39^.

### Pathogenicity and transmission of H10 viruses in ducks

Duckling determined to be negative for H9N2, H5N1, H6N1, H3N6, and H4N6 subtype avian influenza viruses were obtained and used, 4-week-old ducks (10/group) were intranasally inoculated with 100 μl viruses at a dilution of 10^6 ^EID_50_. At 2 dpi, 4-week-old uninfected ducks (6/group) were introduced into each group as direct contacts. Three inoculated ducks from each group were euthanized at 3 dpi and 5 dpi, and three contact ducks from each group were euthanized at 5 dpi. The remaining ducks were monitored daily for clinical signs, were euthanized at 14 dpi, and serum was collected for HI assays. The HI assay was conducted according to the WHO manual on animal Influenza^39^.

### Virulence of H10 viruses in Balb/c mice

To determine the virulence of the H10 viruses in mammals, Balb/c mice were infected intranasally. Female, 4-week-old Balb/c mice were purchased from Merialvital Co., Beijing. Studies were initiated when mice were 5 weeks old. Mice were anesthetized with isoflurane prior to intranasal inoculation. Each mouse received 1 × 10^6 ^EID_50_ per 50 μl intranasally (14 mice/group). Negative control mice received PBS buffer. Mice were monitored daily for 10 dpi for clinical signs of disease, including lack of grooming, presence of rough coat, respiratory distress or discharge, neurological signs, body weight loss, and survival. A scoring system was used and mice were euthanized if a moribund state was reached. Three mice/group were sacrificed at 3 dpi and 5 dpi, respectively, to determine virus titers in the samples from the nasal turbinate, lung, liver, spleen, kidney and brain. Surviving mice (8/group) were euthanized at 10 dpi. The sera were collected for HI assay. For virus titrations, tissues were weighed and homogenized with a tungsten carbide bead (200 mm) in 0.01 M PBS to produce a concentration of 0.1 g/ml (wt/vol) homogenate, which was oscillated 70 times at 1/s for 2 min in a Tissue Lyser apparatus^55^. After centrifugation at 12,000 rpm for 10 min, 100 μl aliquots of the supernatants were collected and serially diluted into 9-day-old embyonated unvaccinated eggs. Virus titers were subsequently measured in EID_50_ assays. Lung samples were also fixed in 10% formalin and subsequently embedded in paraffin for Hematoxylin and eosin (H & E) staining. All the experiments were carried out in triplicate and the means of results were used for optimization.

### Statistical analysis

All the data were graphed and statistical analyses were performed using the Prism 6 software (GraphPad, La Jolla, CA). Comparisons between two groups’ means were carried out with a two-tailed Student *t* test, whereas multiple comparisons were carried out by an analysis of variance (one-way ANOVA method). The differences were considered statistically significant at *P* values of <0.05 or <0.01.

## Additional Information

**How to cite this article**: Zhang, M. *et al.* Characterization of the Pathogenesis of H10N3, H10N7, and H10N8 Subtype Avian Influenza Viruses Circulating in Ducks. *Sci. Rep.* **6**, 34489; doi: 10.1038/srep34489 (2016).

## Supplementary Material

Supplementary Information

## Figures and Tables

**Table 1:** H10 subtype AIV isolates.

Isolates	Subtype	Abbr.	Viral titers (EID_50_/ml)	IVPI*	Genomic Accession Number
A/duck/Shanghai/602/2009	H10N8	SH602	1.00 × 10^8^	0.39	KU921391 (PB2); KU921394 (PB1); KU921397 (PA); KU921400 (HA); KU921403 (NP); KU921406 (NA); KU921409 (M); KU921412 (NS)
A/duck/Fujian/1761/2010	H10N3	FJ1761	5.62 × 10^8^	1.60	KU921392 (PB2); KU921395 (PB1); KU921398 (PA); KU921401 (HA); KU921404 (NP); KU921407 (NA); KU921410 (M); KU921413 (NS)
A/duck/Shanxi/3180/2010	H10N7	SX3180	3.16 × 10^8^	1.27*	KU921393 (PB2); KU921396 (PB1); KU921399 (PA); KU921402 (HA); KU921405 (NP); KU921408 (NA); KU921411 (M); KU921414 (NS)

^*^Note: Exceptionally viruses of H10 subtype have given IVPI’s marginally in excess of 1.20 and would, according to the European Union definition, be classified as highly pathogenic irrespective of the amino acid motif at the cleavage site^39^.

## References

[b1] Kim H. R. *et al.* Characterization of H10 subtype avian influenza viruses isolated from wild birds in South Korea. Veterinary microbiology 161, 222–228 (2012). 22841407 10.1016/j.vetmic.2012.07.014

[b2] Vijaykrishna D. *et al.* The recent establishment of North American H10 lineage influenza viruses in Australian wild waterfowl and the evolution of Australian avian influenza viruses. Journal of virology 87, 10182–10189 (2013). 23864623 10.1128/JVI.03437-12 PMC3753992

[b3] Ma C. *et al.* Emergence and evolution of H10 subtype influenza viruses in poultry in China. Journal of virology 89, 3534–3541 (2015). 25589662 10.1128/JVI.03167-14 PMC4403437

[b4] Wood G. W., Banks J., Strong I., Parsons G. & Alexander D. J. An avian influenza virus of H10 subtype that is highly pathogenic for chickens, but lacks multiple basic amino acids at the haemagglutinin cleavage site. Avian pathology : journal of the W.V.P.A 25, 799–806 (1996). 18645899 10.1080/03079459608419182

[b5] Englund L. & Hard af Segerstad, C. Two avian H10 influenza A virus strains with different pathogenicity for mink (Mustela vison). Archives of virology 143, 653–666 (1998). 9638139 10.1007/s007050050321

[b6] Sun L. *et al.* Lack of exposure of H10N8 avian influenza virus among veterinarians in Guangdong Province, China. J Med Virol 87, 2018–2020 (2015). 25980313 10.1002/jmv.24268

[b7] Zhang H. *et al.* A human-infecting H10N8 influenza virus retains a strong preference for avian-type receptors. Cell Host Microbe 17, 377–384 (2015). 25766296 10.1016/j.chom.2015.02.006 PMC4359746

[b8] Mikami T. *et al.* Isolation of ortho- and paramyxoviruses from migrating feral ducks in Hokkaido. Brief Report. Archives of virology 74, 211–217 (1982). 7165509 10.1007/BF01314714

[b9] Wisedchanwet T. *et al.* Influenza A virus surveillance in live-bird markets: first report of influenza A virus subtype H4N6, H4N9, and H10N3 in Thailand. Avian Dis 55, 593–602 (2011). 22312979 10.1637/9681-020811-Reg.1

[b10] Darveau A., Seidah N. G., Chretien M. & Lecomte J. Peptide mapping of 125I-labelled membrane protein of influenza viruses by reverse-phase high-performance liquid chromatography. J Virol Methods 4, 77–85 (1982). 7076781 10.1016/0166-0934(82)90078-7

[b11] Arzey G. G. *et al.* Influenza virus A (H10N7) in chickens and poultry abattoir workers, Australia. Emerging infectious diseases 18, 814–816 (2012). 22516302 10.3201/eid1805.111852 PMC3358073

[b12] Krog J. S. *et al.* Influenza A(H10N7) virus in dead harbor seals, Denmark. Emerging infectious diseases 21, 684–687 (2015). 25811098 10.3201/eid2104.141484 PMC4378493

[b13] Wu H. *et al.* Novel reassortant H10N7 avian influenza viruses isolated from chickens in Eastern China. Journal of clinical virology : the official publication of the Pan American Society for Clinical Virology 65, 58–61 (2015). 25766990 10.1016/j.jcv.2015.02.007

[b14] Karunakaran D., Hinshaw V., Poss P., Newman J. & Halvorson D. Influenza A outbreaks in Minnesota turkeys due to subtype H10N7 and possible transmission by waterfowl. Avian Dis 27, 357–366 (1983). 6870718

[b15] Englund L. Studies on influenza viruses H10N4 and H10N7 of avian origin in mink. Veterinary microbiology 74, 101–107 (2000). 10799782 10.1016/s0378-1135(00)00170-x

[b16] Woolcock P. R., Shivaprasad H. L. & De Rosa M. Isolation of avian influenza virus (H10N7) from an emu (Dromaius novaehollandiae) with conjunctivitis and respiratory disease. Avian Dis 44, 737–744 (2000). 11007030

[b17] Serena Beato M., Terregino C., Cattoli G. & Capua I. Isolation and characterization of an H10N7 avian influenza virus from poultry carcasses smuggled from China into Italy. Avian pathology: journal of the W.V.P.A 35, 400–403 (2006). 16990150 10.1080/03079450600920992

[b18] Abolnik C. *et al.* Phylogenetic analysis of influenza A viruses (H6N8, H1N8, H4N2, H9N2, H10N7) isolated from wild birds, ducks, and ostriches in South Africa from 2007 to 2009. Avian Dis 54, 313–322 (2010). 20521652 10.1637/8781-040109-Reg.1

[b19] Zohari S., Neimanis A., Harkonen T., Moraeus C. & Valarcher J. F. Avian influenza A(H10N7) virus involvement in mass mortality of harbour seals (Phoca vitulina) in Sweden, March through October 2014. Euro Surveill 19 (2014). 10.2807/1560-7917.es2014.19.46.20967 25425511

[b20] Bodewes R. *et al.* Avian Influenza A(H10N7) virus-associated mass deaths among harbor seals. Emerging infectious diseases 21, 720–722 (2015). 25811303 10.3201/eid2104.141675 PMC4378483

[b21] Bodewes R. *et al.* Seroprevalence of Antibodies against Seal Influenza A(H10N7) Virus in Harbor Seals and Gray Seals from the Netherlands. PLoS One 10, e0144899 (2015). 26658347 10.1371/journal.pone.0144899 PMC4684379

[b22] Wu H. *et al.* Multiple amino acid substitutions involved in the adaptation of avian-origin influenza A (H10N7) virus in mice. Archives of virology (2015). 10.1007/s00705-015-2722-6 26699787

[b23] Bodewes R. *et al.* Spatiotemporal analysis of the genetic diversity of seal influenza A(H10N7) virus, Northwestern Europe. Journal of virology (2016). 10.1128/JVI.03046-15 PMC4836327 26819311

[b24] Wang N. *et al.* Complete genome sequence of an H10N5 avian influenza virus isolated from pigs in central China. Journal of virology 86, 13865–13866 (2012). 23166264 10.1128/JVI.02687-12 PMC3503105

[b25] Chen H. *et al.* Clinical and epidemiological characteristics of a fatal case of avian influenza A H10N8 virus infection: a descriptive study. Lancet 383, 714–721 (2014). 24507376 10.1016/S0140-6736(14)60111-2

[b26] Su S. *et al.* First evidence of H10N8 Avian influenza virus infections among feral dogs in live poultry markets in Guangdong province, China. Clin Infect Dis 59, 748–750 (2014). 24812294 10.1093/cid/ciu345

[b27] Qi W. *et al.* Genesis of the novel human-infecting influenza A(H10N8) virus and potential genetic diversity of the virus in poultry, China. Euro Surveill 19 (2014). 10.2807/1560-7917.es2014.19.25.20841 24993558

[b28] Hu M. *et al.* Coexistence of Avian Influenza Virus H10 and H9 Subtypes among Chickens in Live Poultry Markets during an Outbreak of Infection with a Novel H10N8 Virus in Humans in Nanchang, China. Jpn J Infect Dis 68, 364–369 (2015). 25766608 10.7883/yoken.JJID.2014.377

[b29] Zhu W. *et al.* Dual E627K and D701N mutations in the PB2 protein of A(H7N9) influenza virus increased its virulence in mammalian models. Sci Rep 5, 14170 (2015). 26391278 10.1038/srep14170 PMC4585756

[b30] Deng G. *et al.* Genetics, Receptor Binding, and Virulence in Mice of H10N8 Influenza Viruses Isolated from Ducks and Chickens in Live Poultry Markets in China. Journal of virology 89, 6506–6510 (2015). 25855738 10.1128/JVI.00017-15 PMC4474319

[b31] Garcia-Sastre A. & Schmolke M. Avian influenza A H10N8–a virus on the verge? Lancet 383, 676–677 (2014). 24508318 10.1016/S0140-6736(14)60163-X

[b32] Zhang T. *et al.* Human infection with influenza virus A(H10N8) from live poultry markets, China, 2014. Emerging infectious diseases 20, 2076–2079 (2014). 25425075 10.3201/eid2012.140911 PMC4257803

[b33] Song M. S. *et al.* Unique determinants of neuraminidase inhibitor resistance among N3, N7, and N9 avian influenza viruses. J Virol (2015). 10.1128/JVI.01514-15 PMC4621141 26292325

[b34] Gao R. *et al.* Human infection with a novel avian-origin influenza A (H7N9) virus. N Engl J Med 368, 1888–1897 (2013). 23577628 10.1056/NEJMoa1304459

[b35] Bogs J. *et al.* Highly pathogenic H5N1 influenza viruses carry virulence determinants beyond the polybasic hemagglutinin cleavage site. PLoS One 5, e11826 (2010). 20676399 10.1371/journal.pone.0011826 PMC2910732

[b36] Liu Q. *et al.* Emergence of a novel drug resistant H7N9 influenza virus: evidence based clinical potential of a natural IFN-alpha for infection control and treatment. Expert review of anti-infective therapy 12, 165–169 (2014). 24350808 10.1586/14787210.2014.870885

[b37] Andreas L. B., Eddy M. T., Chou J. J. & Griffin R. G. Magic-angle-spinning NMR of the drug resistant S31N M2 proton transporter from influenza A. Journal of the American Chemical Society 134, 7215–7218 (2012). 22480220 10.1021/ja3003606 PMC3342473

[b38] Zielecki F. *et al.* Virulence determinants of avian H5N1 influenza A virus in mammalian and avian hosts: role of the C-terminal ESEV motif in the viral NS1 protein. Journal of virology 84, 10708–10718 (2010). 20686040 10.1128/JVI.00610-10 PMC2950580

[b39] WHO http://www.wpro.who.int/emerging_diseases/documents/docs/manualonanimalaidiagnosisandsurveillance.pdf. (2002).

[b40] Song W. *et al.* The K526R substitution in viral protein PB2 enhances the effects of E627K on influenza virus replication. Nat Commun 5, 5509 (2014). 25409547 10.1038/ncomms6509 PMC4263149

[b41] Li X. L. *et al.* Risk Distribution of Human Infections with Avian Influenza H7N9 and H5N1 virus in China. Sci Rep 5, 18610 (2015). 26691585 10.1038/srep18610 PMC4686887

[b42] Duan L. *et al.* Influenza virus surveillance in migratory ducks and sentinel ducks at Poyang Lake, China. Influenza Other Respir Viruses 5 Suppl 1, 65–68 (2011). 21751456

[b43] Shao Q. *et al.* Function of duck RIG-I in induction of antiviral response against IBDV and avian influenza virus on chicken cells. Virus Res 191, 184–191 (2014). 25128465 10.1016/j.virusres.2014.07.028

[b44] Chen Y. *et al.* Duck RIG-I CARD Domain Induces the Chicken IFN-beta by Activating NF-kappaB. Biomed Res Int 2015, 348792 (2015). 25918711 10.1155/2015/348792 PMC4396137

[b45] Klingeborn B., Rott E. L. Juntti R. Rockborn N. & An G. avian influenza A virus killing a mammalian species-the mink. Arch. Virol. 86, 347–351 (1985). 4062564 10.1007/BF01309839

[b46] Zohari S., Kiss M. G. Belák I. Berg S. & Full M. genome comparison and characterization of avian H10 viruses with different pathogenicity in mink (Mustela vison) reveals genetic and functional differences in the non-structural gene. Virol. J 7, 145 (2010). 20591155 10.1186/1743-422X-7-145 PMC2909961

[b47] Qi L., Davis P. L. Schwartzman A. S. Chertow L. M. Baxter D. S. Scherler D. Hartshorn K. Slemons K. L. Walters R. D. Kash K. A. & Taubenberger J. C. JK Contemporary avian influenza A virus subtype H1, H6, H7, H10, and H15 hemagglutinin genes encode a mammalian virulence factor similar to the 1918 pandemic virus H1 hemagglutinin. mBio 5, e02116 (2014). 25406382 10.1128/mBio.02116-14 PMC4251996

[b48] Ramos I. *et al.* Hemagglutinin Receptor Binding of a Human Isolate of Influenza A(H10N8) Virus. Emerging infectious diseases 21, 1197–1201 (2015). 26079843 10.3201/eid2107.141755 PMC4480385

[b49] Mok C. K. *et al.* Amino acid residues 253 and 591 of the PB2 protein of avian influenza virus A H9N2 contribute to mammalian pathogenesis. Journal of virology 85, 9641–9645 (2011). 21734052 10.1128/JVI.00702-11 PMC3165745

[b50] Mok C. K. *et al.* Amino acid substitutions in polymerase basic protein 2 gene contribute to the pathogenicity of the novel A/H7N9 influenza virus in mammalian hosts. Journal of virology 88, 3568–3576 (2014). 24403592 10.1128/JVI.02740-13 PMC3957932

[b51] Chen G. W. *et al.* Genomic Signatures for Avian H7N9 Viruses Adapting to Humans. PLoS One 11, e0148432 (2016). 26845764 10.1371/journal.pone.0148432 PMC4742285

[b52] Li Q. *et al.* Adaptation of a natural reassortant H5N2 avian influenza virus in mice. Veterinary microbiology 172, 568–574 (2014). 25037995 10.1016/j.vetmic.2014.06.018

[b53] Li Q. *et al.* Adaptive mutations in PB2 gene contribute to the high virulence of a natural reassortant H5N2 avian influenza virus in mice. Virus Res 210, 255–263 (2015). 26315686 10.1016/j.virusres.2015.08.017

[b54] Hoffmann E., Stech J., Guan Y., Webster R. G. & Perez D. R. Universal primer set for the full-length amplification of all influenza A viruses. Archives of virology 146, 2275–2289 (2001). 11811679 10.1007/s007050170002

[b55] Chen H. *et al.* All-in-one bacmids: an efficient reverse genetics strategy for influenza A virus vaccines. Journal of virology 88, 10013–10025 (2014). 24942589 10.1128/JVI.01468-14 PMC4136356

