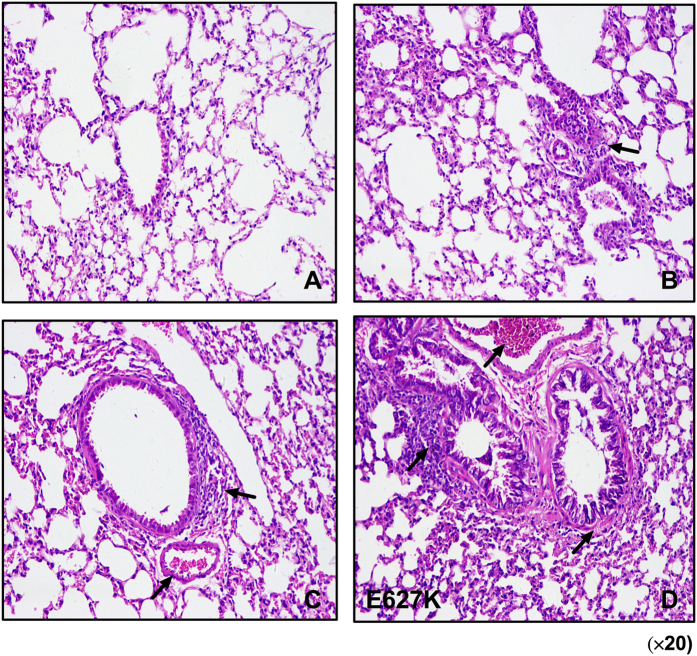# Erratum: Characterization of the Pathogenesis of H10N3, H10N7, and H10N8 Subtype Avian Influenza Viruses Circulating in Ducks

**DOI:** 10.1038/srep44343

**Published:** 2017-03-23

**Authors:** Miaomiao Zhang, Xingxing Zhang, Kaidi Xu, Qiaoyang Teng, Qinfang Liu, Xuesong Li, Jianmei Yang, Jianqing Xu, Hongjun Chen, Xiaoyan Zhang, Zejun Li

Scientific Reports 6: Article number: 3448910.1038/srep34489; published online: 09 28 2016; updated: 03 23 2017

In this Article, Figures 1–5 are incorrect. The correct Figures 1, 2, 3, 4, 5 appear below as Figure 1, Figure 2, Figure 3, Figure 4 and Figure 5 respectively. The Figure legends are correct.

## Figures and Tables

**Figure 1:**
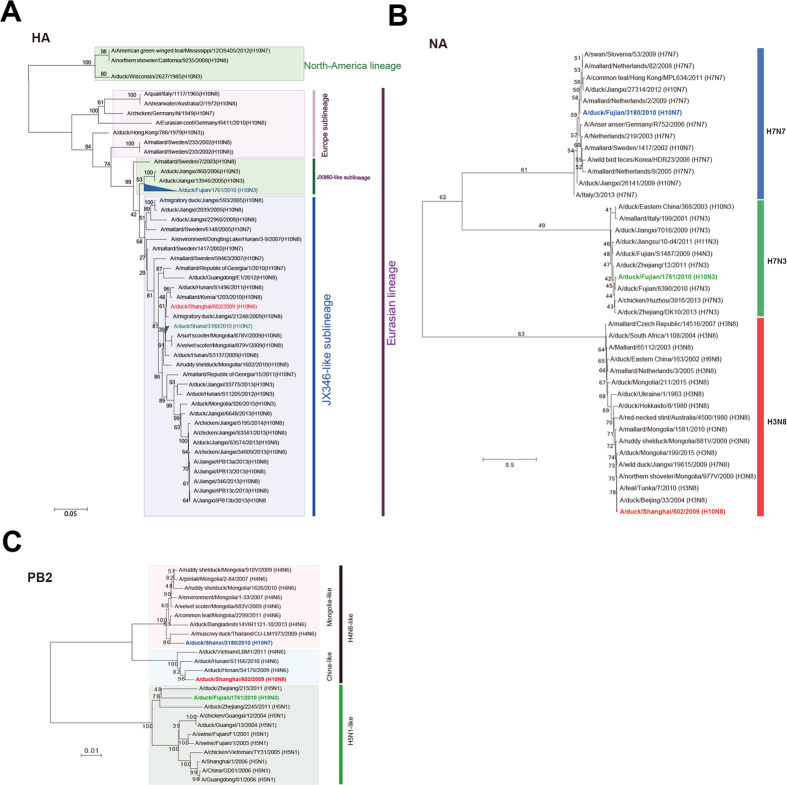


**Figure 2:**
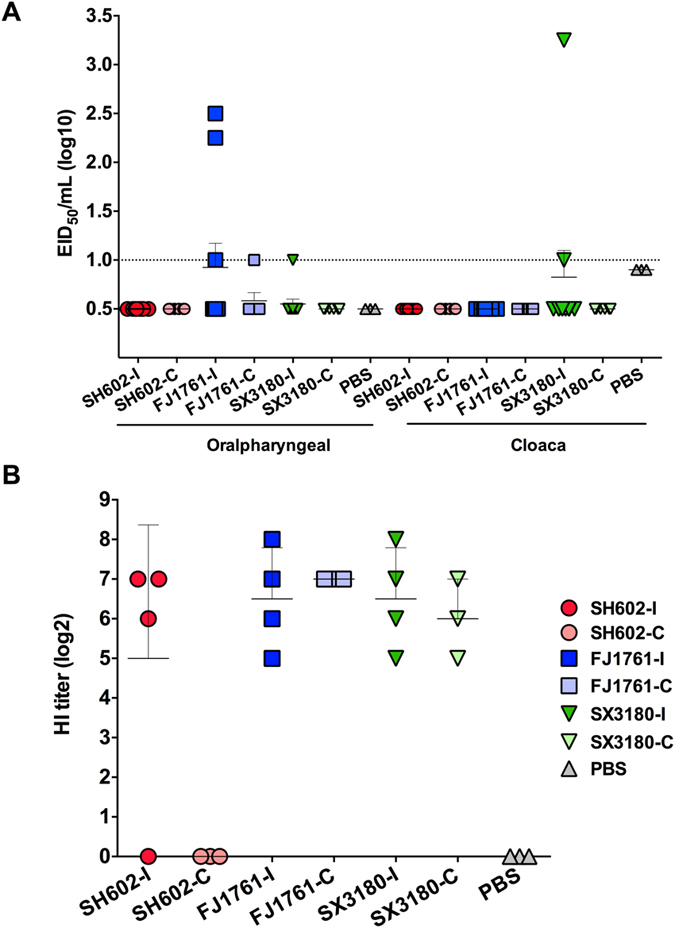


**Figure 3:**
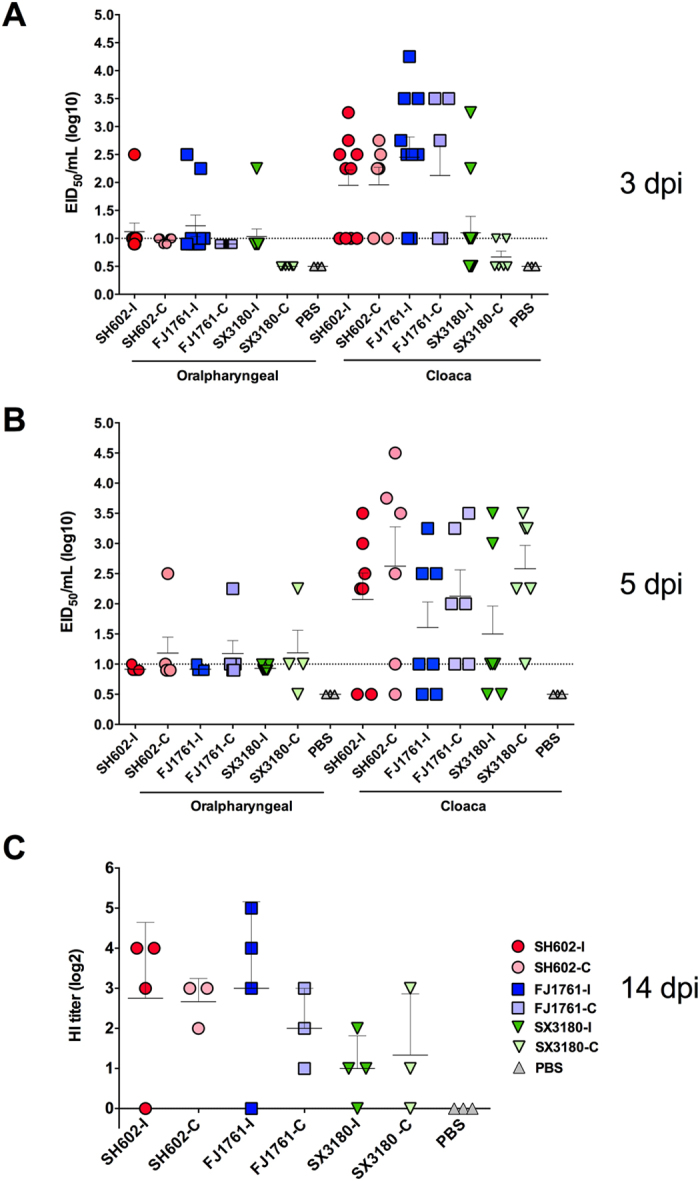


**Figure 4:**
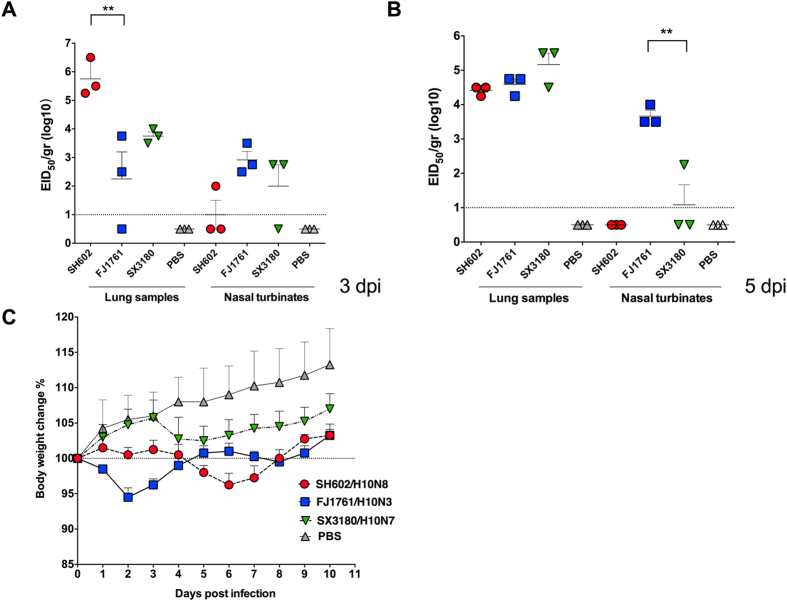


**Figure 5:**