# Supplementary material for: Characterization of the Pathogenesis of H10N3, H10N7, and H10N8 Subtype Avian Influenza Viruses Circulating in Ducks
Source: Sci Rep. 2016 Sep 28;6:34489. doi: 10.1038/srep34489 (PMC5039634; doi:10.1038/srep34489)
Supplement: Supplementary Information [file srep34489-s1.pdf]

Supplementary information

Characterization on Pathogenesis of H10 Subtype Avian Influenza Viruses Circulating in Ducks

Miaomiao Zhang [1,2], Xingxing Zhang [2], Kaidi Xu [2], Qiaoyang Teng [2], Qinfang Liu [2], Xuesong Li [2],Jianmei Yang [2], Jianqing Xu [1], Hongjun Chen [2*], Xiaoyan Zhang [1*], Zejun Li [2*]

Figure. 1 **Phylogenetic tree of HA and NA sequences of H10 subtype AIVs**

The phylogenetic tree was generated with MEGA6 software, which was based on the complete sequence of PB1 (A), PA (B), NP (C), M (D), NS (E). Different color of each segment represents supposed reassortment patterns for the H10 viruses.

PB1

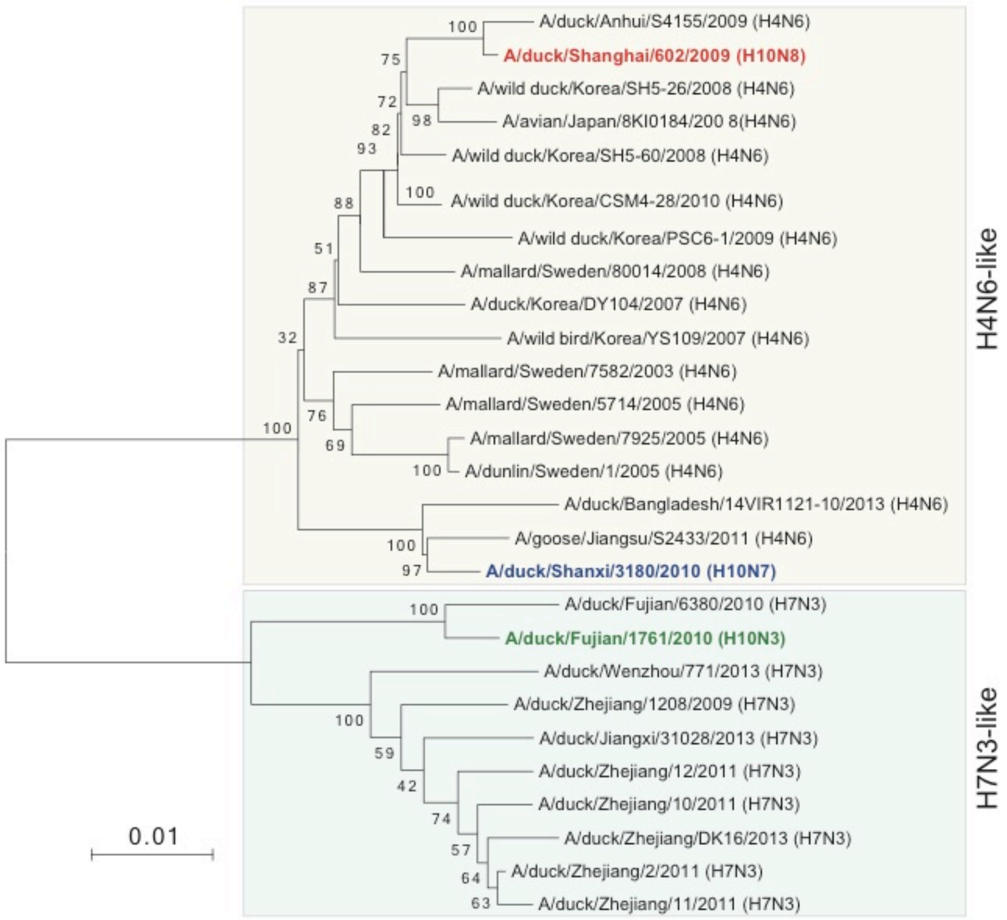

A/duck/Anhui/S4155/2009 (H4N6)
A/duck/Shanghai/602/2009 (H10N8)
A/wild duck/Korea/SH5-26/2008 (H4N6)
A/avian/Japan/8KI0184/200 8(H4N6)
A/wild duck/Korea/SH5-60/2008 (H4N6)
A/wild duck/Korea/CSM4-28/2010 (H4N6)
A/wild duck/Korea/PSC6-1/2009 (H4N6)
A/mallard/Sweden/80014/2008 (H4N6)
A/duck/Korea/DY104/2007 (H4N6)
A/wild bird/Korea/YS109/2007 (H4N6)
A/mallard/Sweden/7582/2003 (H4N6)
A/mallard/Sweden/5714/2005 (H4N6)
A/mallard/Sweden/7925/2005 (H4N6)
A/dunlin/Sweden/1/2005 (H4N6)
A/duck/Bangladesh/14VIR1121-10/2013 (H4N6)
A/goose/Jiangsu/S2433/2011 (H4N6)
A/duck/Shanxi/3180/2010 (H10N7)
H4N6-like
A/duck/Fujian/6380/2010 (H7N3)
A/duck/Fujian/1761/2010 (H10N3)
A/duck/Wenzhou/771/2013 (H7N3)
A/duck/Zhejiang/1208/2009 (H7N3)
A/duck/Jiangxi/31028/2013 (H7N3)
A/duck/Zhejiang/12/2011 (H7N3)
A/duck/Zhejiang/10/2011 (H7N3)
A/duck/Zhejiang/DK16/2013 (H7N3)
A/duck/Zhejiang/2/2011 (H7N3)
A/duck/Zhejiang/11/2011 (H7N3)
H7N3-like
0.01

PA

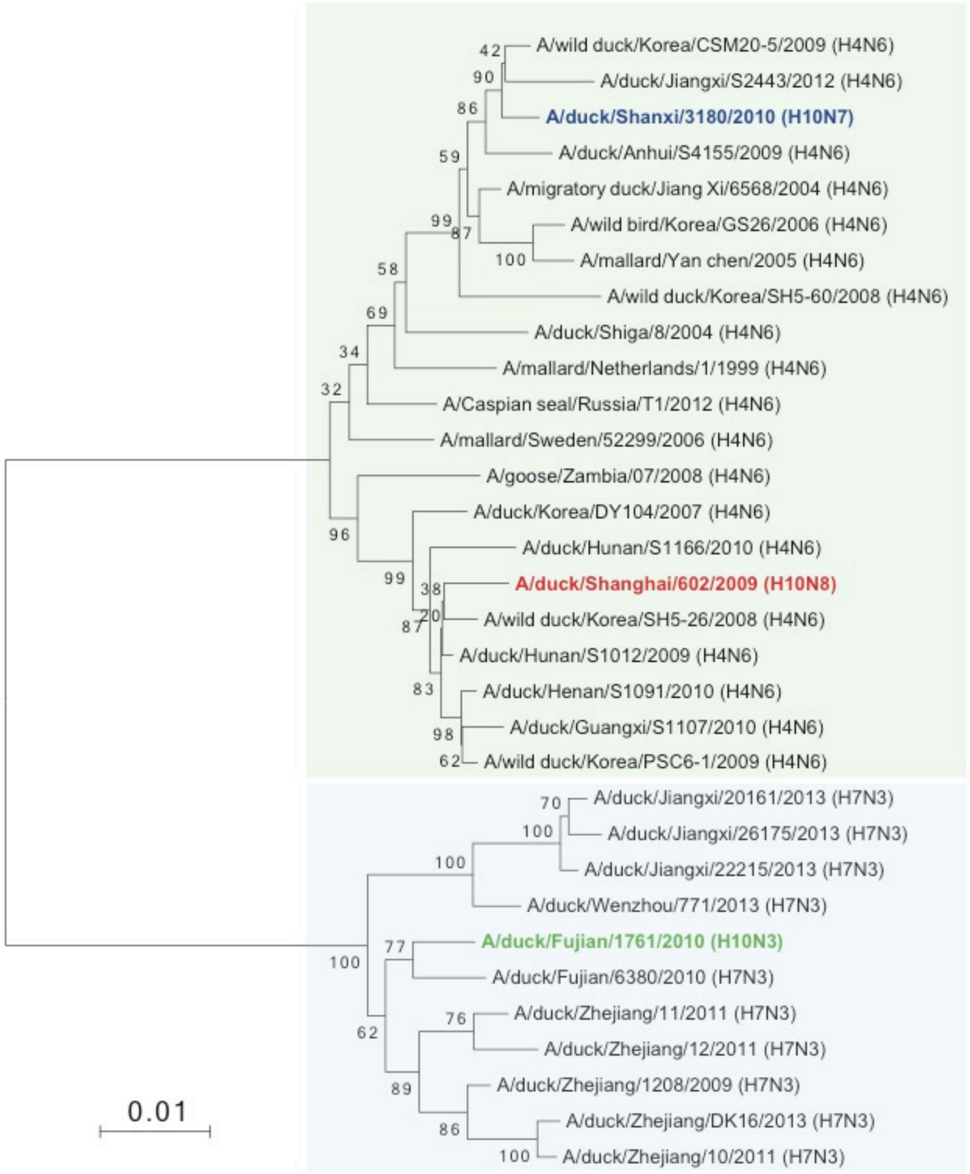
A/wild duck/Korea/CSM20-5/2009 (H4N6)
A/duck/Jiangxi/S2443/2012 (H4N6)
A/duck/Shanxi/3180/2010 (H10N7)
A/duck/Anhui/S4155/2009 (H4N6)
A/migratory duck/Jiang Xi/6568/2004 (H4N6)
A/wild bird/Korea/GS26/2006 (H4N6)
A/mallard/Yan chen/2005 (H4N6)
A/wild duck/Korea/SH5-60/2008 (H4N6)
A/duck/Shiga/8/2004 (H4N6)
A/mallard/Netherlands/1/1999 (H4N6)
A/Caspian seal/Russia/T1/2012 (H4N6)
A/mallard/Sweden/52299/2006 (H4N6)
A/goose/Zambia/07/2008 (H4N6)
A/duck/Korea/DY104/2007 (H4N6)
A/duck/Hunan/S1166/2010 (H4N6)
A/duck/Shanghai/602/2009 (H10N8)
A/wild duck/Korea/SH5-26/2008 (H4N6)
A/duck/Hunan/S1012/2009 (H4N6)
A/duck/Henan/S1091/2010 (H4N6)
A/duck/Guangxi/S1107/2010 (H4N6)
A/wild duck/Korea/PSC6-1/2009 (H4N6)
A/duck/Jiangxi/20161/2013 (H7N3)
A/duck/Jiangxi/26175/2013 (H7N3)
A/duck/Jiangxi/22215/2013 (H7N3)
A/duck/Wenzhou/771/2013 (H7N3)
A/duck/Fujian/1761/2010 (H10N3)
A/duck/Fujian/6380/2010 (H7N3)
A/duck/Zhejiang/11/2011 (H7N3)
A/duck/Zhejiang/12/2011 (H7N3)
A/duck/Zhejiang/1208/2009 (H7N3)
A/duck/Zhejiang/DK16/2013 (H7N3)
A/duck/Zhejiang/10/2011 (H7N3)
0.01

NP

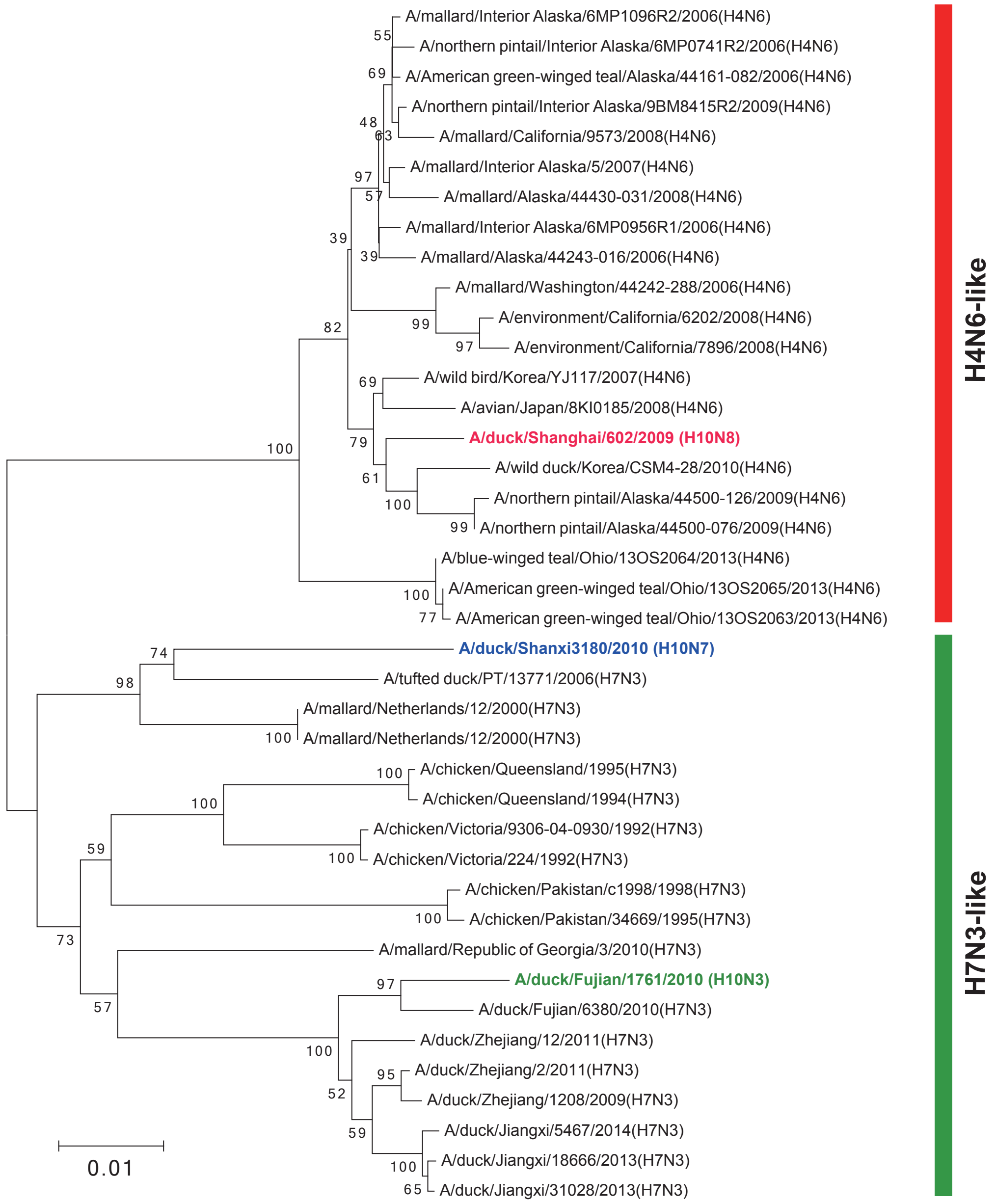
A/mallard/Interior Alaska/6MP1096R2/2006(H4N6)
A/northern pintail/Interior Alaska/6MP0741R2/2006(H4N6)
A/American green-winged teal/Alaska/44161-082/2006(H4N6)
A/northern pintail/Interior Alaska/9BM8415R2/2009(H4N6)
A/mallard/California/9573/2008(H4N6)
A/mallard/Interior Alaska/5/2007(H4N6)
A/mallard/Alaska/44430-031/2008(H4N6)
A/mallard/Interior Alaska/6MP0956R1/2006(H4N6)
A/mallard/Alaska/44243-016/2006(H4N6)
A/mallard/Washington/44242-288/2006(H4N6)
A/environment/California/6202/2008(H4N6)
A/environment/California/7896/2008(H4N6)
A/wild bird/Korea/YJ117/2007(H4N6)
A/avian/Japan/8KI0185/2008(H4N6)
A/duck/Shanghai/602/2009 (H10N8)
A/wild duck/Korea/CSM4-28/2010(H4N6)
A/northern pintail/Alaska/44500-126/2009(H4N6)
A/northern pintail/Alaska/44500-076/2009(H4N6)
A/blue-winged teal/Ohio/13OS2064/2013(H4N6)
A/American green-winged teal/Ohio/13OS2065/2013(H4N6)
A/American green-winged teal/Ohio/13OS2063/2013(H4N6)
A/duck/Shanxi3180/2010 (H10N7)
A/tufted duck/PT/13771/2006(H7N3)
A/mallard/Netherlands/12/2000(H7N3)
A/mallard/Netherlands/12/2000(H7N3)
A/chicken/Queensland/1995(H7N3)
A/chicken/Queensland/1994(H7N3)
A/chicken/Victoria/9306-04-0930/1992(H7N3)
A/chicken/Victoria/224/1992(H7N3)
A/chicken/Pakistan/c1998/1998(H7N3)
A/chicken/Pakistan/34669/1995(H7N3)
A/mallard/Republic of Georgia/3/2010(H7N3)
A/duck/Fujian/1761/2010 (H10N3)
A/duck/Fujian/6380/2010(H7N3)
A/duck/Zhejiang/12/2011(H7N3)
A/duck/Zhejiang/2/2011(H7N3)
A/duck/Zhejiang/1208/2009(H7N3)
A/duck/Jiangxi/5467/2014(H7N3)
A/duck/Jiangxi/18666/2013(H7N3)
A/duck/Jiangxi/31028/2013(H7N3)
H4N6-like
H7N3-like
0.01

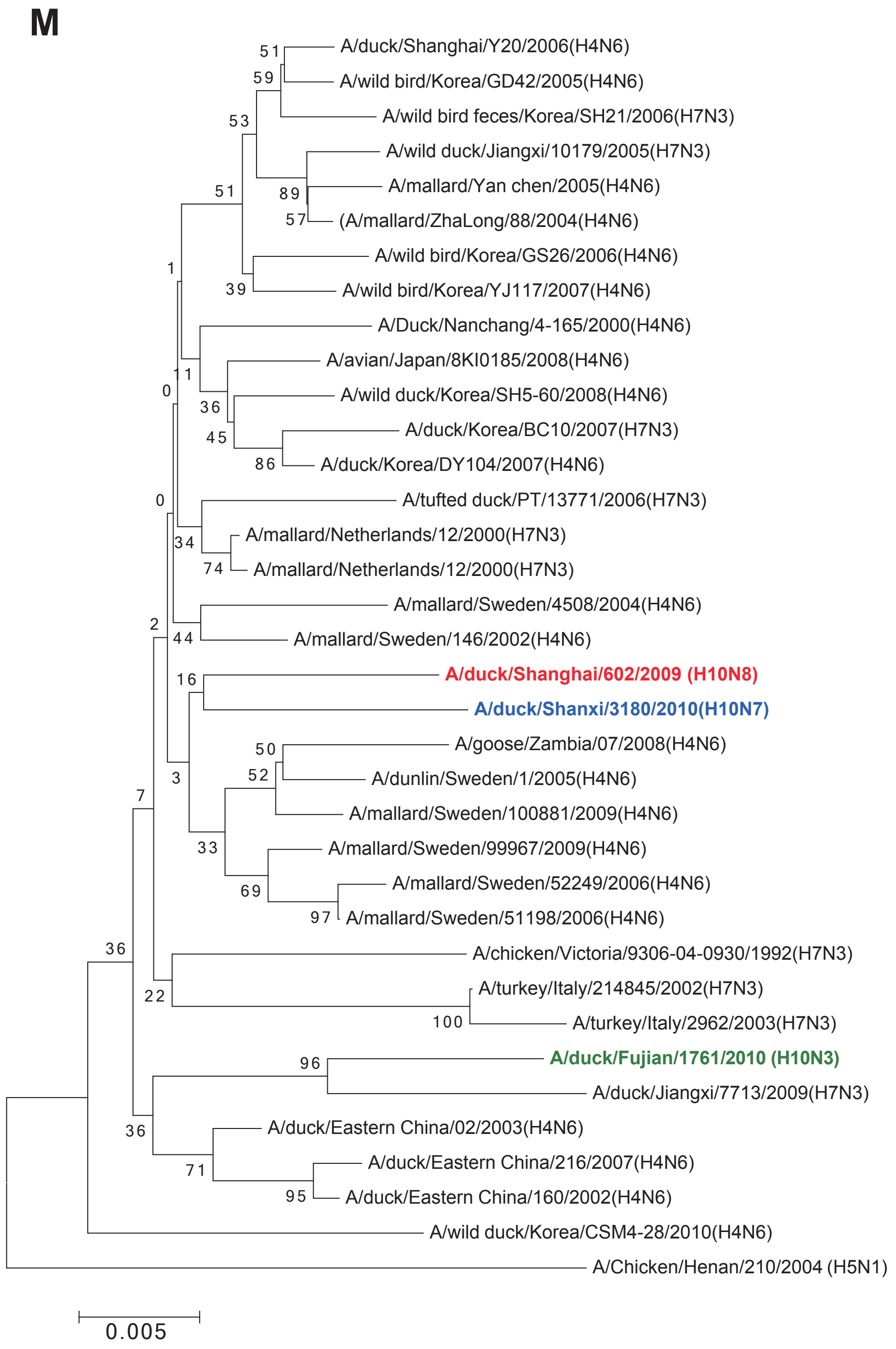
M
A/duck/Shanghai/Y20/2006(H4N6)
A/wild bird/Korea/GD42/2005(H4N6)
A/wild bird feces/Korea/SH21/2006(H7N3)
A/wild duck/Jiangxi/10179/2005(H7N3)
A/mallard/Yan chen/2005(H4N6)
(A/mallard/ZhaLong/88/2004(H4N6)
A/wild bird/Korea/GS26/2006(H4N6)
A/wild bird/Korea/YJ117/2007(H4N6)
A/Duck/Nanchang/4-165/2000(H4N6)
A/avian/Japan/8KI0185/2008(H4N6)
A/wild duck/Korea/SH5-60/2008(H4N6)
A/duck/Korea/BC10/2007(H7N3)
A/duck/Korea/DY104/2007(H4N6)
A/tufted duck/PT/13771/2006(H7N3)
A/mallard/Netherlands/12/2000(H7N3)
A/mallard/Netherlands/12/2000(H7N3)
A/mallard/Sweden/4508/2004(H4N6)
A/mallard/Sweden/146/2002(H4N6)
A/duck/Shanghai/602/2009 (H10N8)
A/duck/Shanxi/3180/2010(H10N7)
A/goose/Zambia/07/2008(H4N6)
A/dunlin/Sweden/1/2005(H4N6)
A/mallard/Sweden/100881/2009(H4N6)
A/mallard/Sweden/99967/2009(H4N6)
A/mallard/Sweden/52249/2006(H4N6)
A/mallard/Sweden/51198/2006(H4N6)
A/chicken/Victoria/9306-04-0930/1992(H7N3)
A/turkey/Italy/214845/2002(H7N3)
A/turkey/Italy/2962/2003(H7N3)
A/duck/Fujian/1761/2010 (H10N3)
A/duck/Jiangxi/7713/2009(H7N3)
A/duck/Eastern China/02/2003(H4N6)
A/duck/Eastern China/216/2007(H4N6)
A/duck/Eastern China/160/2002(H4N6)
A/wild duck/Korea/CSM4-28/2010(H4N6)
A/Chicken/Henan/210/2004 (H5N1)
0.005

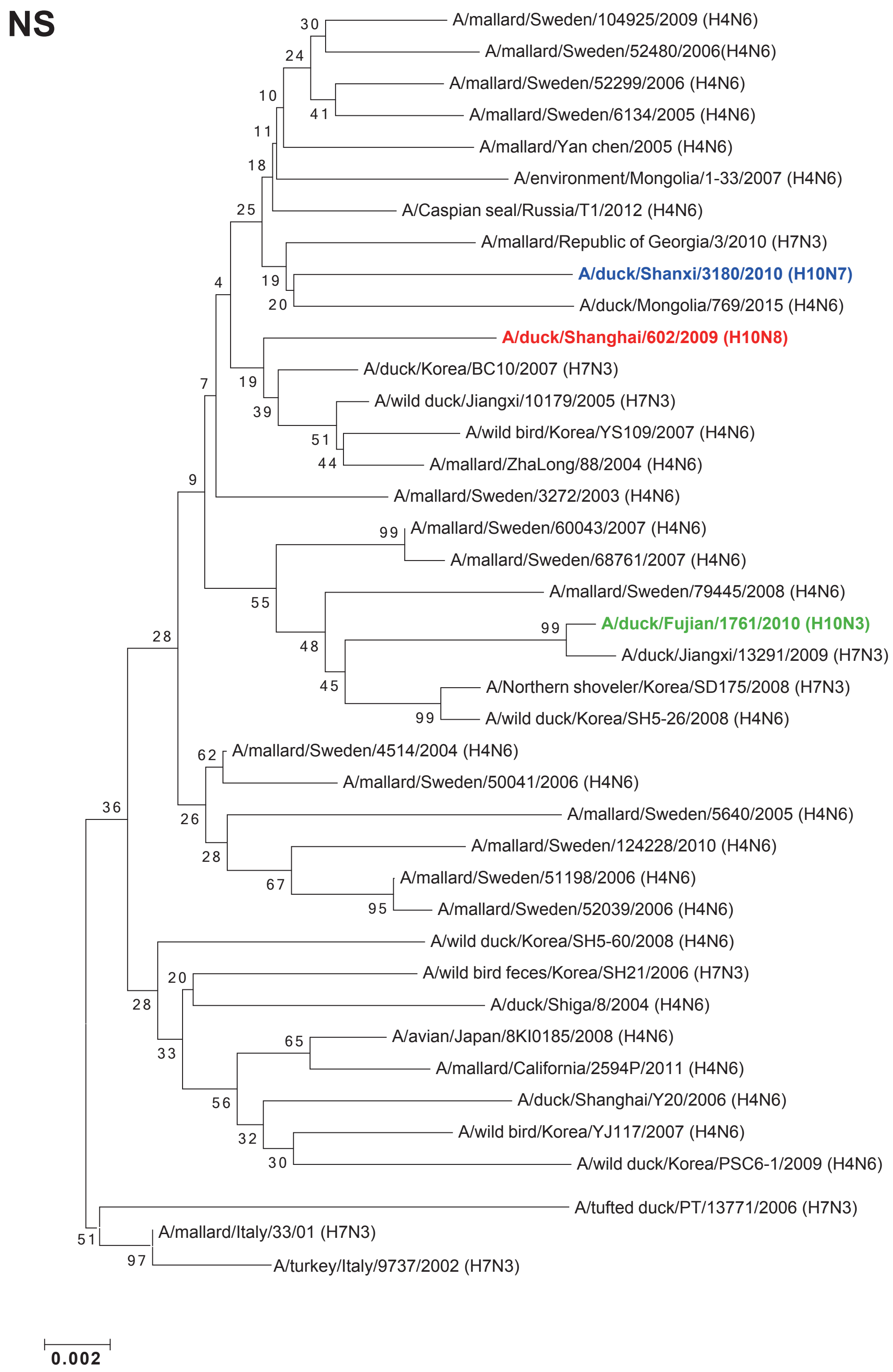

NS
A/mallard/Sweden/104925/2009 (H4N6)
A/mallard/Sweden/52480/2006(H4N6)
A/mallard/Sweden/52299/2006 (H4N6)
A/mallard/Sweden/6134/2005 (H4N6)
A/mallard/Yan chen/2005 (H4N6)
A/environment/Mongolia/1-33/2007 (H4N6)
A/Caspian seal/Russia/T1/2012 (H4N6)
A/mallard/Republic of Georgia/3/2010 (H7N3)
A/duck/Shanxi/3180/2010 (H10N7)
A/duck/Mongolia/769/2015 (H4N6)
A/duck/Shanghai/602/2009 (H10N8)
A/duck/Korea/BC10/2007 (H7N3)
A/wild duck/Jiangxi/10179/2005 (H7N3)
A/wild bird/Korea/YS109/2007 (H4N6)
A/mallard/ZhaLong/88/2004 (H4N6)
A/mallard/Sweden/3272/2003 (H4N6)
A/mallard/Sweden/60043/2007 (H4N6)
A/mallard/Sweden/68761/2007 (H4N6)
A/mallard/Sweden/79445/2008 (H4N6)
A/duck/Fujian/1761/2010 (H10N3)
A/duck/Jiangxi/13291/2009 (H7N3)
A/Northern shoveler/Korea/SD175/2008 (H7N3)
A/wild duck/Korea/SH5-26/2008 (H4N6)
A/mallard/Sweden/4514/2004 (H4N6)
A/mallard/Sweden/50041/2006 (H4N6)
A/mallard/Sweden/5640/2005 (H4N6)
A/mallard/Sweden/124228/2010 (H4N6)
A/mallard/Sweden/51198/2006 (H4N6)
A/mallard/Sweden/52039/2006 (H4N6)
A/wild duck/Korea/SH5-60/2008 (H4N6)
A/wild bird feces/Korea/SH21/2006 (H7N3)
A/duck/Shiga/8/2004 (H4N6)
A/avian/Japan/8KI0185/2008 (H4N6)
A/mallard/California/2594P/2011 (H4N6)
A/duck/Shanghai/Y20/2006 (H4N6)
A/wild bird/Korea/YJ117/2007 (H4N6)
A/wild duck/Korea/PSC6-1/2009 (H4N6)
A/tufted duck/PT/13771/2006 (H7N3)
A/mallard/Italy/33/01 (H7N3)
A/turkey/Italy/9737/2002 (H7N3)
0.002

1
